# Climatic variability and morbidity and mortality associated with particulate matter

**DOI:** 10.11606/S1518-8787.2017051006952

**Published:** 2017-11-07

**Authors:** Poliany Cristiny de Oliveira Rodrigues, Samya de Lara Pinheiro, Washington Junger, Eliane Ignotti, Sandra de Souza Hacon

**Affiliations:** I Universidade do Estado de Mato Grosso. Cáceres, MT, Brasil; II Aria do Brasil. Rio de Janeiro, RJ, Brasil; III Universidade do Estado do Rio de Janeiro. Rio de Janeiro, RJ, Brasil; IV Escola Nacional de Saúde Pública. Fundação Oswaldo Cruz. Rio de Janeiro, RJ, Brasil

**Keywords:** Particulate Matter, adverse effects, Air Pollutants, Cardiovascular Diseases, epidemiology, Risk Factors, Seasons, Ecological Studies, Time Series Studies, Material Particulado, efeitos adversos, Poluentes do Ar, Doenças Cardiovasculares, epidemiologia, Fatores de Risco, Estações do Ano, Estudos Ecológicos, Estudos de Séries Temporais

## Abstract

**OBJECTIVE:**

The objective of this study has been to analyze whether fine particulate matter (PM_2.5_), as well as its synergistic effect with maximum temperature, humidity, and seasons, is associated with morbidity and mortality from cardiovascular diseases.

**METHODS:**

This is an ecological study of time series. We have used as outcomes the daily death and hospitalization records of adults aged 45 years and over from 2009 to 2011 of the municipalities of Cuiabá and Várzea Grande, State of Mato Grosso, Brazil. We have used Poisson regression using generalized additive models, assuming a significance level of 5%. The model has been controlled for temporal trend, seasonality, average temperature, humidity, and season effects. Daily concentrations of PM_2.5_ (particulate material with aerodynamic diameter less than 2.5 micrometers) have been obtained by converting the values of optical aerosol thickness. Maximum temperature, humidity, and seasons have been separately included in the model as dummy variables for the analysis of the synergistic effect of PM_2.5_ with morbidity and mortality from cardiovascular disease. We have calculated the percentage increase of relative risk (%RR) of deaths and hospitalizations for the linear increase of 10 μg/m^3^ of PM_2.5_.

**RESULTS:**

Between 2009 and 2011, the increase in PM_2.5_ was associated with a %RR 2.28 (95%CI 0.53–4.06) for hospitalizations on the same day of exposure and RR% 3.57 (95%CI 0.82–6.38) for deaths with a lag of three days. On hot days, %RR 4.90 (95%CI -0.61–9.38) was observed for deaths. No modification of the effect of PM_2.5_ was observed for maximum temperature in relation to hospitalizations. On days with low humidity, %RR was 5.35 (95%CI -0.20–11.22) for deaths and 2.71 (95%CI -0.39–5.92) for hospitalizations. In the dry season, %RR was 2.35 (95%CI 0.59–4.15) for hospitalizations and 3.43 (95%CI 0.58–6.35) for deaths.

**CONCLUSIONS:**

The PM_2.5_ is associated with morbidity and mortality from cardiovascular diseases and its effects may be potentiated by heat and low humidity and during the dry season.

## RESUMO

**OBJETIVO:**

Analisar se o material particulado fino (PM_2,5_), bem como seu efeito sinérgico com a temperatura máxima, a umidade e as estações do ano estão associados à morbimortalidade por doenças cardiovasculares.

**MÉTODOS:**

Estudo ecológico de séries temporais. Foram utilizados como desfechos os registros diários de óbito e internação em adultos com 45 anos ou mais de idade para os anos de 2009 a 2011 nos municípios de Cuiabá e Várzea Grande, Mato Grosso, Brasil. Utilizou-se regressão de Poisson via modelos aditivos generalizados, assumindo-se um nível de significância de 5%. O modelo foi controlado para tendência temporal, sazonalidade, temperatura média, umidade e efei**t**os de calendário. Concentrações diárias de PM_2,5_ (material particulado com diâmetro aerodinâmico inferior a 2,5 micrômetros) foram obtidas por meio da conversão dos valores da espessura ótica de aerossóis. Temperatura máxima, umidade e estações do ano foram incluídas separadamente ao modelo como variáveis indicadoras para análise do efeito sinérgico do PM_2,5_ com a morbimortalidade por doenças cardiovasculares. Foi calculado o aumento percentual de risco relativo (%RR) dos óbitos e internações para o aumento linear de 10 μg/m^3^ de PM_2,5_.

**RESULTADOS:**

Entre 2009 e 2011, o aumento de PM_2,5_ foi associado a um %RR 2,28 (IC95% 0,53–4,06) para internações no mesmo dia de exposição e %RR 3,57 (IC95% 0,82–6,38) para óbitos com uma defasagem de três dias. Em dias quentes, observou-se %RR 4,90 (IC95% -0,61–9,38) para óbitos. Não foi observada modificação de efeito do PM_2,5_ pela temperatura máxima relacionada as internações. Em dias com baixa umidade, o %RR foi 5,35 (IC95% -0,20–11,22) para óbitos e 2,71 (IC95% -0,39–5,92) para internações. Na estação seca, o %RR foi 2,35 (IC95% 0,59–4,15) para internações e 3,43 (IC95% 0,58–6,35) para óbitos.

**CONCLUSÕES:**

O PM_2,5_ está associado à morbimortalidade por doenças cardiovasculares e seus efeitos podem ser potencializados pelo calor, pela baixa umidade e durante a estação seca.

## INTRODUCTION

There is evidence that exposure to fine particulate matter (PM_2.5_) is associated with an increased risk of mortality, hospitalization, and exacerbation of cardiovascular diseases (CD). The magnitude of the reported effects, however, is related to the composition, amount, entryway, transport capacity, and deposition of PM in the organism. The PM_2.5_ shows greater toxicity because of its multielementarity and because it can both reach the lower portions of the respiratory tract and directly contact the bloodstream^5^.

Geographic location and climatic seasonality may also influence the magnitude of adverse effects of PM on human health. Some studies have shown that climate can modify the association of PM_2.5_ with morbidity and mortality. Although temperature is the most studied parameter^8^ ^,^ ^10^ ^,^ ^17^ ^,^ ^23^, some authors have observed that humidity, atmospheric pressure, and seasons can also act as modifiers of the effect of PM on health events^14^ ^,^ ^16^.

In the Brazilian Amazon and Cerrado, in particular, climatic phenomena intensify the health effects related to atmospheric pollution. In these regions, some ecological studies show that PM is associated with increased morbidity and mortality from respiratory^6^ ^,^ ^22^ and cardiovascular diseases^12^ ^,^ ^19^ in children and the elderly, especially during the dry season, in which accidental or intentional burning of pastures, sugarcane, and forest occurs. However, the modifying effects of climatic seasonality of the region on the morbidity and mortality associated with air pollution have not been investigated yet.

The characterization of the effects of PM_2.5_, as well as their combined effect with climatic variables, can better explain the heterogeneity of the effects observed in the region and it can subsidize mitigation and control actions that are more effective and adequate to the needs of the local population in relation to air pollution. The objective of this study was to analyze whether fine particulate matter (PM_2.5_), as well as its synergistic effect with maximum temperature, humidity, and seasons are associated with morbidity and mortality from cardiovascular diseases.

## METHODS

This is an ecological study of time series of daily records of deaths and hospitalizations for cardiovascular diseases. We selected individuals aged 45 years and over who lived in the municipalities of Cuiabá and Várzea Grande and who were hospitalized and died from CD (Chapter IX of the Tenth Revision of the International Classification of Diseases [ICD-10], codes I00 to I99) between April 23, 2009 and December 31, 2011. The time series from 2009 to 2011, comprised of 983 days, was selected because of the availability of optical aerosol thickness data that enabled us to estimate PM_2.5_.

The municipalities of Cuiabá and Várzea Grande, located in the State of Mato Grosso, Brazil, form a conurbation that groups a population of approximately 820,000 inhabitants, which is approximately 90% of the total population of the metropolitan region of the Cuiabá River Valley. The population aged 45 years and over represents approximately 60% of the total population of the cities^a^.

The climate of the region is limited to two remarkable seasons: the dry and rainy season. Between the months of May to October, the dry period, the prevalence of fires is historically high in the region. The most intense rainy season corresponds to the months of November to April. The cities are well known for their strong daily heat of 32°C, on average. The average maximum temperature reaches 41°C in the hottest months^b^. Cardiovascular diseases appear as the first cause of general mortality between 2009 and 2011, with an average mortality rate of 135.35 deaths per 100,000 inhabitants, following the mortality profile of the State^c^. In the same period, diseases of the circulatory system were the fifth cause of hospitalization in the State of Mato Grosso and the third cause of hospitalization in Cuiabá and Várzea Grande, with an average hospitalization rate of 46.25 admissions per 10,000 inhabitants^c^.

The records on mortality and hospitalization for CD used as outcomes were obtained, respectively, from the Mortality Information System (SIM/SUS) and the Hospital Information System (SIH/SUS) databases. Information of the daily averages of temperature and humidity came from the National Institute of Meteorology (INMET).

Aerosol Optical Depth (AOD) data were obtained from the Cuiabá-Miranda station (Latitude: -15.43; Longitude: -56.01) available on the Aerosol Robotic Network (AERONET) website. Estimates of PM_2.5_ were generated by converting the AOD values (500 nanometers), using a method developed and validated for the Brazilian Amazon and Cerrado^d^. The calculation basis of this method allows us to obtain values of concentrations of PM_2.5_ very close to the data measured by air monitoring stations^2^ and it has been used to supply the lack of atmospheric pollution information in some areas^1^ ^,^ ^19^. Although the measurements obtained represent average values for the entire atmospheric column with a spatial resolution of up to 10 km, the validation^d^ showed that the conversion is adequate for the deforestation arc and the estimated PM_2.5_ obtained can be used as an alternative for studies on the impact of PM on human health^2^.

A time series regression was performed, and we constructed explanatory models to count the hospitalizations and deaths from CD over time. The time series method has been widely applied in ecological epidemiological studies, mainly in the evaluation of the acute effect, because it presents better performance in the analysis of the linear effects of air pollution on mortality from CD^10^ ^,^ ^13^.

The generalized additive model (GAM) class was used, with Poisson regression, assuming a significance level of 5%. The temporal trend and the seasonality of the series were controlled introducing the following variables: days of the week and a natural cubic spline of the time with nine degrees of freedom. For the meteorological variables, we used the natural cubic spline of the average temperature and relative humidity, with three and two degrees of freedom, respectively. In the modeling process, we sought to reduce the Akaike information criterion (AIC) and improve partial autocorrelation (PACF).

We chose to use the GAM model because we do not need to define a priori the relationships and structures between the health indicator and the explanatory variables. In addition, we used the ARES2 library available in the R application as a tool to define these relationships. The Poisson regression model, with logarithmic link function, was chosen because the mortality data obtained by counting have a Poisson probability distribution^7^ ^,^ ^13^.

The percentage increase of relative risk (%RR) of deaths and hospitalizations for CD was calculated. We investigated associations of current day exposures with single lag and the cumulative effect of up to ten days using a polynomial distributed lag model. The 10-day lag period was selected to allow a more accurate estimate of the effects of PM_2.5_ on mortality and hospitalizations for CD, as these effects may not occur immediately or on the same day as the exposure^5^ ^,^ ^18^ ^,^ ^23^. The %RR corresponds to the linear increase of 10 μg/m^3^in the levels of PM_2.5_.

The synergistic effect between PM_2.5_ and maximum temperature, humidity, and season was tested by stratifying the final models built for hospitalizations and deaths. The stratification method consists in the insertion of a dummy variable in the final models, observing the new characteristics of the same model with stratification^7^. We chose to use stratification with this method to use fewer parameters and to offer a simple and quantitative comparison of the estimated effects of pollutants in the different strata in relation to other methods to detect the modifying effect^10^ ^,^ ^13^. Three dummy variables were included in the model: (i) 90th percentile of the maximum temperature (37.9°C), (ii) 10th percentile of relative humidity (54.5%), and (iii) dry (May – October) and rainy season (January to March, November and December). The statistical significance of the differences between the estimates of the effect among the strata established by the dummy variables was determined by calculating the 95% confidence interval (95% CI) and the p-value^7^.

The analyses were performed in the R 3.0.2 application using the Ares2 library^7^. This study has been approved by the Ethics Committee of the Escola Nacional de Saúde Pública (CAAE 18634613.0.0000.5240).

## RESULTS

During the study period, 8,610 hospitalizations and 3,024 deaths were recorded. Particulate material and relative humidity showed a heterogeneous distribution during the year, presenting standard deviations (SD) of 15.66 μg/m^3^ and 11.35%, respectively. The maximum values of the average and maximum temperatures are almost three times higher than their respective minimum values (Table 1).

**Table 1:** Descriptive statistics of the environmental and health variables under study. Cuiabá and Várzea Grande, State of Mato Grosso, Brazil, 2009 to 2011. (n = 983)

Variable	Days with no information	Average	Standard deviation	Minimum	Maximum
Death from CD (n)	0	3.08	1.74	0.00	9.00
Hospitalization for CD (n)	0	8.76	3.55	1.00	20.00
PM_2.5_ (μg/m^3^)	55*	17.07	15.66	0.10	172.30
Average temperature (°C)	0	26.45	3.04	11.44	33.44
Maximum temperature (°C)	0	33.69	3.88	13.30	42.30
Relative humidity (%)	0	70.71	11.35	35.00	97.00

CD: cardiovascular diseases; PM_2.5_: fine particulate matter

* Empty spaces are common in series from remote sensing because of cloudy days, clouds, and smoke. They corresponded to only 0.17% of the series and in non-grouped days.

Average temperature and maximum temperature showed a direct correlation of approximately 0.25 with PM_2.5_. Humidity presented an inverse correlation with all variables, being strongly associated with maximum temperature (-0.600). Deaths and hospitalizations for CD presented a linear correlation below 0.100 with the meteorological and pollution variables (Table 2).

**Table 2:** Pearson correlation matrix for the environmental and health variables under study. Cuiabá and Várzea Grande, State of Mato Grosso, Brazil, 2009 to 2011.

Variable	Deaths from CD	Hospitalizations for CD	PM_2.5_	Average temperature	Maximum temperature	Humidity
Deaths from CD	1					
Hospitalizations for CD	0.069*	1				
PM_2.5_	0.011	0.009	1			
Average temperature	-0.097*	-0.057	0.262*	1		
Maximum temperature	-0.066	-0.009	0.250*	0.850*	1	
Humidity	-0.024	-0.039	-0.189*	-0.383*	-0.600*	1

CD: cardiovascular diseases; PM_2.5_: fine particulate matter

* p < 0.05

**Figure 1:**
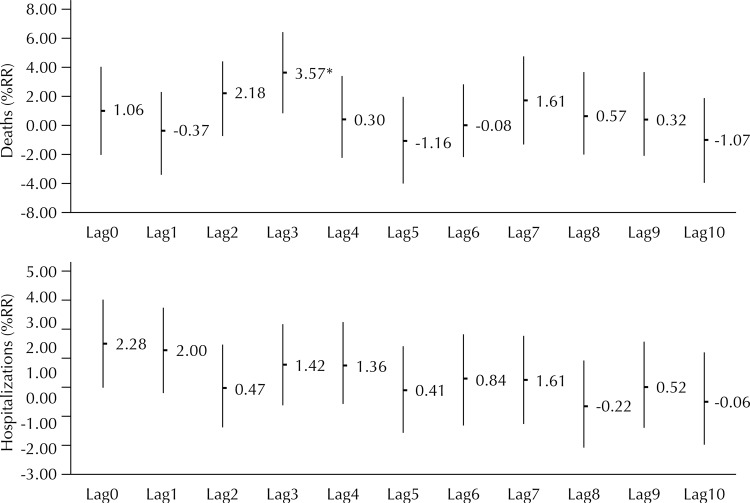
Percent relative risk (%RR) for hospitalization and mortality from cardiovascular diseases related to increments of 10 μg/m3 of PM2.5 by single lag. Cuiabá and Várzea Grande, State of Mato Grosso, Brazil, 2009 to 2011.

In the modeling by the simple lag method, we observed a cyclical effect of reduction and increase every three days, both for hospitalizations and for deaths associated with the linear increase of PM2.5. However, the effect was more acute for hospitalizations. The hospitalizations for CD presented %RR 2.28 (95%CI 0.53–4.06) for the same day of exposure (Lag0) and %RR 2.00 (95%CI 0.31–3.71) for the sigle lag of one day (Lag1). Mortality presented %RR 3.57 (95%CI 0.82–6.38) for the sigle lag of three days (Lag3) (Figure 1).

* p ≤ 0.05

**Figure 2:**
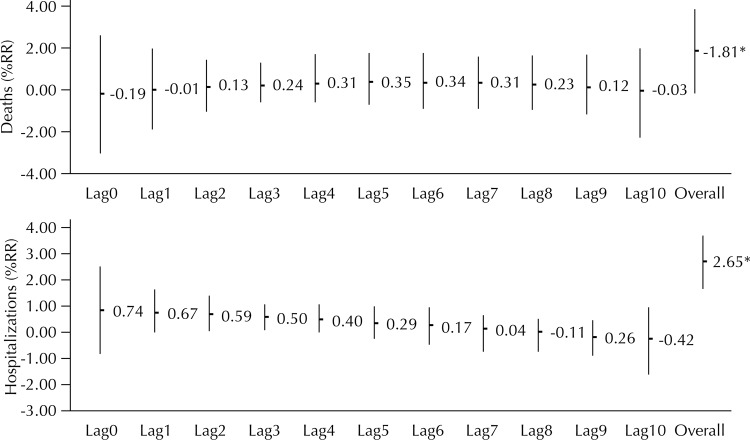
Percent relative risk (%RR) for hospitalization and mortality from cardiovascular diseases related to increments of 10 μg/m3 of PM2.5 by distributed lag. Cuiabá and Várzea Grande, State of Mato Grosso, Brazil, 2009 to 2011.

The accumulated %RR for the period of ten days was 1.81 (95%CI 0.03–3.61) for deaths and 2.64 (95%CI 1.60–3.69) for hospitalizations related to the increase in PM_2.5_. The distributed lag method reinforced the acute effect pattern for hospitalizations. There was an increase in %RR of mortality from the second day of exposure and decrease in %RR for hospitalizations throughout the days (Figure 2).

* p ≤ 0.05

In the dry season, approximately 50% of the observations of maximum temperature were between 34.8°C and 39.10°C, while approximately 50% of the relative humidity data were between 66% and 51%. The rainy season had approximately 50% of the observations between 33°C and 36.4°C, while approximately 50% of the relative humidity data were between 77% and 65%. The average PM_2.5_ was 18.71 μg/m^3^ (SD = 19.59 μg/m^3^) and 14.88 μg/m^3^ (SD = 7.18 μg/m^3^) in the dry and rainy seasons, respectively.

The results of the analyses on the modifying effect show that temperature, humidity, and season can present synergistic effects of both deaths and hospitalizations for CD related to PM_2.5_. The %RR of mortality from CD related to PM_2.5_ was 4.90 (95%CI -0.61–9.38) on days whose maximum temperature was above 37.9°C. The %RR of mortality and hospitalizations for CD related to PM_2.5_ were 5.35 (95%CI -0.20–11.22) and 2.71 (95%CI -0.39–5.92), respectively, on days with relative humidity below 54.5%. During the dry season, we observed %RR of 3.43 (95%CI 0.58–6.35) for mortality and %RR 2.35 (95%CI 0.59–4.15) for hospitalizations. We observed no modification of the effect of PM_2.5_ by the maximum temperature related to hospitalizations (Table 3).

**Table 3:** Percent relative risk (%RR) and confidence intervals (CI) of the synergistic effects of season, maximum temperature, and humidity for hospitalization and mortality from cardiovascular diseases related to increments of 10 μg/m3 of PM2.5 by single lag. Cuiabá and Várzea Grande, State of Mato Grosso, Brazil, 2009 to 2011.

Variable		Hospitalizations		Deaths	
	
		%RR	95%CI	%RR	95%CI
Season	Dry	2.35*	0.59–4.15	3.43*	0.58–6.35
	Wet	1.05	-5.37–7.91	-4.53	-11.95–3.50
Maximum temperature	> 37.9°C	0.95	-1.85–3.82	4.90*	0.61–9.38
	< 37.9°C	-1.80	-4.95–1.45	1.55	-3.49–6.86
Humidity	> 54.5%	0.57	-2.75–4.02	2.05	-3.68–8.14
	< 54.5%	2.71*	-0.39–5.92	5.35*	-0.20–11.22

* p ≤ 0.05

## DISCUSSION

The results of this study reinforce findings from the international^5^ and Brazilian^11^ ^,^ ^20^ literature by pointing out the relationship between PM_2.5_ and the increased risk of hospitalization and mortality from CD in adults aged 45 years and over. The adverse effects of PM on hospitalizations for CD coincided with the day of the increase of pollutant (Lag0) and the risk decreased on subsequent days, while the effects of PM on mortality from CD increased after the increase of pollutant, culminating in mortality mainly after the third day of exposure (lag3). This relationship corroborates the literature, which indicates that cardiovascular diseases are related to more outcomes that are nonspecific and with many-associated causes^5^. Thus, although both outcomes are related to more severe cases, hospitalizations are acute, whereas mortality may be associated with the complication of the diseases^20^.

Heat had a synergistic effect with PM_2.5_ on mortality from CD in this study. This synergism between temperature and mortality from CD is still controversial in the scientific literature; however, some authors have observed that adverse effects of particulate matter may be more apparent in warmer seasons^8^ ^,^ ^23^ and at higher temperatures^10^ ^,^ ^17^. Ren and Tong^17^ have observed %RR of 2.32 (95%CI -0.26–4.97) for hospitalizations for CD and 6.95 (95%CI 0.95–13.33) for mortality from CD related to PM_10_ on days with temperature above 27°C in Brisbane, Australia, between 1996 and 2001. Meng et al.^10^ have observed %RR of 1.57% (95%CI 0.69–2.46) for cardiovascular mortality related to PM_10_ on days with temperature above 30°C (> 95th percentile) for eight cities in China between 2001 and 2008, whose RR% was 3.19% (95%CI 2.55–3.84) for the city of Wuhan. In the United States, an ecological study carried out for 207 cities has observed an increase in mortality from CD related to PM_2.5_ in cities with higher average temperatures^8^.

Modification of the effects of PM_2.5_ by high temperatures on human health may be related to the direct or indirect response of the organism to heat stress^18^. Body thermoregulation is directly linked to the circulatory regulation of an individual. In this way, hot days can overload the body temperature-regulating system and change the physiological response to toxic agents, increasing individual vulnerability to the effects of PM^4^ ^,^ ^17^. On warmer days, a greater exposure to air pollution may occur because of the propensity to keep windows open or to spend more time outdoors^23^. Some studies suggest that high temperatures can also be considered an indirect measure of the composition of urban atmospheric particles^8^, since they are strongly associated with organic and elemental sulfate and carbon^24^, ozone^3^, and the concentration of semi-volatile particles^21^.

Our results showed evidence that the action of PM_2.5_ on hospitalizations and mortality from CD can be exacerbated on days of low relative humidity (below 54.5%). The effects of humidity related to the prevalence of hospitalizations and mortality, as well as their impact on the effects of pollution, are little reported in the literature. Qiu et al.^14^, in China, have observed an increase of 1.67 (95%CI 1.26–2.08) in emergency hospitalizations for ischemic heart diseases related to PM_10_ on days when relative humidity was below 80%. Ravljen et al.^16^, in Slovenia, have identified that the increase in 1% in the average daily humidity decreases the incidence of acute coronary syndrome by approximately 3 ‰ (95%CI -6.00–-1.00). In Cuiabá and Várzea Grande, Brazil, Rodrigues et al.^19^ have observed that high-risk temporal clusters for mortality from CD had higher daily averages of PM_2.5_ and lower daily averages of relative humidity. However, the comparison between these results is difficult because of the great methodological differences.

Seasonality is described by several authors as a modifier of the effects of PM associated with hospitalizations and mortality from CD^8^ ^,^ ^23^ ^,^ ^24^. Some studies point to seasons as indirect measures of temperature, such as Kioumourtzoglou et al.^8^, in the USA, and Staffogia et al.^23^, in Italy, who have found an increase in mortality from CD related to PM_2.5_ and PM_10_, respectively, in the summer. Atmospheric chemical speciation studies suggest that the prevalence of more toxic components may occur at higher concentrations during different times of the year, according to the climatic and pollutant emission characteristics of each site, with rainfall being the most effective mechanism for the deposition of pollutants^15^ ^,^ ^24^ ^,^ ^25^. The findings of this study show that the dry season may increase both hospitalizations and deaths from CD related to PM_2.5_, which is compatible with both hypotheses, firstly because the dry season also had higher temperatures than the rainy season in our database, and secondly because the dry season is also the period with the highest prevalence of fires in the municipalities and in the North and Midwest regions of Brazil.

It should be taken into account that other sources of air pollution may exist, such as vehicular traffic, burning of urban waste, or the transport of air pollutants from biomass burning and industrial emissions from neighboring cities. Although they also had an important influence on morbidity and mortality from CD, these sources were not included in our analyses, as no data were available on these variables. The local climatic condition must also be considered. Cuiabá and Várzea Grande are located in a depression with extensive *Chapadões* (mountain ranges) at their edge and present great periodicity of atmospheric stability (clean sky and low wind speed), which can hinder the dispersion of pollutants and increase the incidence of heat islands and thermal inversion episodes. In addition, the dry season may characterize an indirect measure of the effects of low humidity^19^ ^,^ ^22^, also observed in this study.

As limitations of the study, we can highlight the filling bias inherent to the use of the database on hospitalizations and mortality of the Brazilian Unified Health System (SUS) that may not accurately portray the distribution of the variables studied. However, studies have indicated the reliability of the data contained in health information systems^9^. Another important point is related to the use of estimated values for PM, obtained by remote sensing techniques, which may underestimate or not correspond to the real values of personal exposure; however, its use is feasible and advantageous both for this study and for remote regions that do not have atmospheric monitoring stations^1^ ^,^ ^2^. It is important to mention that the ecological character of our analyses does not allow us to identify any causal effect; that is, the association observed between the aggregates does not mean that the same association occurs at the individual level.

We concluded that PM_2.5_ has an association with hospitalizations and mortality from CD in medium-sized municipalities. Low relative humidity and dry season may increase both hospitalizations and deaths from CD related to PM_2.5_. Heat is related to the increased risk of mortality from CD associated with PM_2.5_.

This is the first study that addresses the interactions of climatic factors with pollution in the Cerrado region. The use of estimated pollution data, obtained by remote sensing techniques, provides results compatible with those found in other ecological studies, indicating that this tool is effective in obtaining the concentrations of PM. The results of this research can be used for an evaluation on the improvement of the quality of the data on mortality from CD and air pollution to base future studies and, mainly, help in the planning of strategies to mitigate the impact of air pollution in human health.

## Footnotes

a Instituto Brasileiro de Geografia e Estatística. IBGE Cidades: indicadores e dados populacionais. Rio de Janeiro (DF); IBGE; 2010 [cited 2013 May 6]. Available from: http://www.ibge.gov.br/cidadesat/xtras/perfil.

b Instituto Nacional de Meteorologia. Dados históricos. Brasília (DF): INMET; 2013 [cited 2013 Jun 15]. Available from: http://www.inmet.gov.br/portal/ php?codmun=510340& search=mato-grosso|cuiaba

c Ministério da Saúde (BR), Departamento de Informática do SUS (DATASUS). Rede Interagencial de Informações para a Saúde - RIPSA. Indicadores e dados básicos – Brasil -2012 – IDB 2012. Brasília (DF); 2012 [cited 2014 May 13]. Available from: http://tabnet.datasus.gov.br/cgi/idb2012/matriz.htm

d Paixão MMA. Propriedades ópticas de aerossóis naturais e de queimadas da Amazônia [dissertation]. São Paulo: Instituto de Física da Universidade de São Paulo; 2011 [cited 2016 Jul 22]. Available from: http://www.teses.usp.br/teses/disponiveis/43/43134/tde-30092011-150552/
